# Mortality Due to Malignant and Non-Malignant Diseases in Korean Professional Emergency Responders

**DOI:** 10.1371/journal.pone.0120305

**Published:** 2015-03-10

**Authors:** Yeon-Soon Ahn, Kyoung Sook Jeong

**Affiliations:** Department of Occupational and Environmental Medicine, Dongguk University, Dongguk University Ilsan Hospital, Goyang, the Republic of Korea; Universität Bochum, GERMANY

## Abstract

**Objective:**

This study was conducted to estimate the cause-specific mortality in male emergency responders (ER), compare with that of Korean men. Mortality was also compared between more experienced firefighters (i.e., firefighters employed ≥20 years and firefighters employed ≥10 to <20 years) and less experienced firefighters and non-firefighters (i.e., firefighters employed <10 years and non-firefighters) to investigate associations between mortality and exposure to occupational hazards.

**Methods:**

The cohort was comprised of 33,442 males who were employed as ERs between 1980 and 2007 and not deceased as of 1991. Work history was merged with the death registry from the National Statistical Office of Korea to follow-up on mortality between 1992 and 2007. Standardized mortality ratios (SMR) for ERs were calculated in reference to the Korean male population. Adjusted relative risks (ARRs) of mortalities for firefighters employed ≥20 years and ≥10 years to <20 years were calculated in reference to non-firefighters and firefighters employed < 10 years.

**Results:**

Overall (SMR=0.43, 95%CI=0.39–0.47) and some kinds of cause-specific mortalities were significantly lower among ERs compared with the Korean male population. No significant increase in mortality was observed across the major ICD-10 classifications among ERs. Mortality due to exposure to smoke, fire, and flames (SMR=3.11, 95% CI=1.87–4.85), however, was significantly increased among ERs. All-cause mortality (ARR=1.46, 95% CI=1.13–1.89), overall cancer mortality (ARR=1.54, 95% CI=1.02–2.31) and mortality of external injury, poisoning and external causes (ARR=3.13, 95% CI=1.80–5.46) were significantly increased among firefighters employed ≥20 years compared to those of non-firefighters and firefighters employed < 10 years.

**Conclusions:**

An increase in mortality due to all cancer and external injury, poisoning, and external causes in firefighters employed ≥20 years compared with non-firefighters and firefighters employed <10 years suggests occupational exposure.

## Introduction

In Korea, responses to emergencies such as fires and requests for emergency medical aid or technical rescues are handled by professional emergency responders (ERs) who belong to the Korea National Emergency Management Agency (NEMA). More than 1.7 million emergency responses (40,928 fires, 1,405,263 requests for emergency medical aid, and 316,776 technical rescues) were recorded in Korea in 2011 [1].

ERs, including firefighters and ambulance and rescue workers, are often exposed to toxic chemicals and physical and biological hazards. ERs also experience significant job-related stress and sleep and circadian disruptions due to shift work schedules. ERs, especially firefighters, are therefore susceptible to work-related injuries and illnesses such as respiratory diseases, ischemic heart diseases, noise-induced hearing loss, infections, musculoskeletal diseases, post-traumatic stress disorders (PTSDs), and cancers.

Many morbidity and mortality studies have been conducted on firefighters and police officers; fewer studies have been conducted on other ERs. In addition, studies that have targeted firefighters have been unable to identify expected fatal work-related diseases such as coronary artery disease and cancers. One meta-analysis of 17 firefighter mortality studies conducted between 1959 and 2001 found no significant association between employment as a firefighter and all-cause, coronary artery disease, cancer, or respiratory disease mortality [2]. This finding suggests that occupational exposures do not contribute significantly to morbidity or mortality among firefighters. An alternate explanation is that these data are influenced by the ‘healthy firefighter effect’.

Considerable differences in job-related exposures between firefighters and other ERs have been shown. Most Korean professional ERs, however, belong to the NEMA and are cross-trained to serve multiple roles that depend on the nature of the emergency. For example, ERs that provide medical aid and assistance with technical rescues during fires are also exposed to the toxic chemicals and physical hazards to which firefighters are exposed In addition, firefighters and other ERs both experience intense job-related stress and shift work schedules [3–5]. All ERs employed by the NEMA, therefore, are exposed to similar occupational hazards [5].

An analysis of work-related illnesses among ERs compensated by the Korean Government Employees Pension Service (GEPS) between 1999 and 2008 revealed that cardiovascular disease was the most common work-related illness (68.2%), followed by certain types of cancer (9.1%), musculoskeletal diseases (5.5%), respiratory diseases (3.6%), and ear diseases (3.6%) [5]. These findings indicate that some diseases occurring among Korean ERs are work-related. However, no mortality studies have been conducted on firefighters or other ERs in the Republic of Korea.

The present study was conducted to investigate the mortality of ERs employed by the NEMA. Mortality among ERs was compared with mortality among the Korean male population. In an attempt to overcome the ‘healthy firefighter effect’ in this cohort study, we also analyzed mortality in firefighters employed ≥20 years and ≥10 to <20 years compared with non-firefighters and firefighters employed <10 years.

## Materials and Methods

### Cohort definition and data collection

The cohort used in this study was comprised of all male professional ERs who were employed by the NEMA for at least one month between 1 January 1980 and 31 December 2007 and not deceased as of 31 December 1991. The follow-up period spanned the first day of employment or 1 January 1992 (whichever occurred later) to the date of death or 31 December 2007 (whichever occurred first). Female ERs, who were relatively young (less than 50 years old), comprised less than 5 percent of the workforce, and were responsible primarily for emergency medical aid, were excluded from the cohort. Vital status was based on employment and mortality records from the National Statistical Office (NSO) of Korea. The NSO estimates that its registry includes more than 99% of the deaths in Korea; cause of death was available beginning in 1992. The NSO records provide the residence registration number (RRN; a unique 13-digit number assigned to all Koreans), the cause of death (International Classification of Diseases, 10^th^ Edition), and the date of death [6]. In Korea, all people must register the birth and death by law. The death certificate including the cause of death was almost written by doctors. So, the cause of death is very reliable.

The NEMA provided the name, RRN, birth date, hiring date, employment termination date, and individual work history for each ER included in the cohort. The work history included information about successive periods at the NEMA and job classification. Nine job titles (firefighting, fire scene investigation, emergency medical aid, technical rescue, driving, ship, plane, computation and communication, and other) were classified into two job categories, firefighters and non-firefighters, based on firefighting experience. Firefighters included all first-line (e.g., pump, ladder, and operation chief) and second-line (e.g., drivers and division chief) firefighting duties. We could not, however, distinguish between different task assignments. Non-firefighters included emergency medical aid and rescue men, ship and plane operators, ambulance drivers, fire scene investigators, computation and communication individuals, administrative staff, and stationary engineers.

Reference mortality rates for the Korean male population were derived from the NSO data for the years 1992 to 2007. The number of mortalities (i.e., the numerator of the mortality rate) was stratified by the type of disease (classified by the ICD-10) and age (in 5-year intervals). The total population (i.e., the denominator of the mortality rate) was obtained from an NSO report of the registered population between the years 1992 and 2007 in which the population numbers were stratified by age (in 5-year intervals).

This work was approved by the Institutional Review Board (IRB) of Dongguk University Ilsan Hospital (2009–1–17). All information used to write this paper was not hospital medical record. The cohort was constructed in 2009. This time, it is just followed the mortality through the mortality registry of Korea National Statistical Office (KNSO). So, IRB of Dongguk University was waived written consent of individual workers or their next of kin. But we received written consent of individual active workers for the prospective follow-up. In Korea the follow-up of the previous constructed cohort before Nov, 2012 is waived written consent, which is based on the premise that researchers protect personal information of all study subjects.

The protocol of this study was neither illegal by Article 18(②(4)) of Personal Information Protection Act in Republic of Korea nor ethical problem to collect data of cause of death.

### Statistical analysis

The Standardized Mortality Ratio (SMR) was calculated using the PAMCOMP program [7]. In total, 377,703 person-years of observations were jointly classified into 12 age groups in 5-year intervals (20–24, 25–29, 30–34,…, 75+), 4 calendar years (1992–1996, 1997–2000, 2001–2004, 2005–2008), and 2 job categories (firefighters vs non-firefighters). Classification was based on a 1-year lag for all malignant and non-malignant deaths. The SMRs and 95% confidence intervals (95% CI) were calculated in reference to the Korean male population by PAMCOMP program automatically. In theory, confidence interval is expressed as following equation at 95% CI.

$${CI}_{SMR}=e^{ln(SMR)\pm Z\left\{SD\left(ln(SMR)\right)\right\}},SD\left(ln(SMR)\right)=1.96$$

The mortality data of Korean male from 1992 to 2007 was opened at website of KNSO.

The adjusted relative risk (ARR) of mortality was calculated using the log-linear model (Poisson regression model) in SPSS (version 12.0; SPSS, Chicago, IL). The relative risk of mortality obtained using Poisson regression with categorical exposure classification (reference group was non-firefighters & firefighters employed <10 years; exposed group were firefighters employed ≥20 years and firefighters employed ≥10 to 20 years, respectively), adjusted for age and calendar year.

We analyzed the cause-specific mortalities in 10 or more cases of death in all ERs to ensure statistical power and analyzed the controversial causes of death such as respiratory diseases and leukemia.

## Results

### Demographics

The study cohort of 33,442 workers was followed for a total of 377,703 person-years. In all ERs, the mean age in 2007 and at first entry to the NEMA was 41.3 and 27.8 years old, respectively. The mean employment duration of all ERs was 15.2 years. In 2007, 84.6% (28,292 workers) of the original cohort was actively employed.

Firefighters comprised 88.1% of the cohort (29,453 workers). The mean job duration was 15.7 years and 11.5 years among firefighters and non-firefighters, respectively. Firefighters were older than non-firefighters (41.8 years old vs. 37.6 years old, respectively) and contributed a higher proportion of retired workers in the cohort compared with non-firefighters (16.6% vs. 6.3%, respectively) (Table 1).

**Table 1:** General characteristics of emergency responders (ERs).

General Characteristics		All ERs		Firefighting Experience			
				Firefighters		Non-firefighters	
		N	%	N	%	N	%
No. of workers		33,442	100.0	29,453	88.1	3,989	11.9
Age in 2007	20–29	2,540	7.6	1,989	6.8	551	13.8
	30–39	13,739	41.1	11,553	39.2	2,186	54.8
	40–49	10,978	32.8	10,088	34.3	890	22.3
	50–59	4,570	13.7	4,271	14.5	299	7.5
	60≤	1,615	4.8	1,552	5.3	63	1.6
	Mean±S.D.*	41.3±9.2		41.8±9.3*		37.6±7.8*	
Year of initial employment	≤1979	3,079	9.2	2,969	10.1	110	2.8
	1980–89	5,709	17.1	5,448	18.5	261	6.5
	1990–99	15,301	45.8	13,408	45.5	1,893	47.5
	2000≤	9,353	28.0	7,628	25.9	1,725	43.2
Age at initial employment	<30	25,045	74.9	22,140	75.2	2,905	72.8
	30–39	7,927	23.7	6,955	23.6	972	24.4
	40 ≤	470	1.4	358	1.2	112	2.8
	Mean±S.D. *	27.8±3.7		27.8±3.6*		28.2±4.1*	
Duration of employment	<10	10,134	30.3	13,864	47.1	2,023	50.7
	10–19	14,959	44.7	10,893	37.0	1,675	42.0
	20 ≤	8,349	25.0	4,696	15.9	291	7.3
	Mean±S.D. *	15.2±8.3		15.7±8.4*		11.5±6.6*	
Employment status	Active	28,292	84.6	24,564	83.4	3,738	93.7
	Retired	5,150	15.4	4,889	16.6	251	6.3

*S*.*D*., standard deviation

*p<0.01

### SMRs for overall and non-malignant deaths

Across all ERs, 308 deaths occurred due to non-malignant causes. The SMRs revealed that mortality due to all causes (SMR: 0.43; 95% CI: 0.39–0.47) and non-malignant diseases (SMR: 0.36; 95% CI: 0.32–0.40) was significantly lower among ERs compared with the Korean male population. Among the 308 non-malignant deaths, 277 occurred in firefighters. Mortality due to all causes (SMR: 0.42; 95% CI: 0.38–0.46) and non-malignant diseases (SMR: 0.35; 95% CI: 0.31–0.39) was significantly lower in firefighters compared with the Korean male population. Similarly, the 41 non-malignant deaths among non-firefighters represented a significant decrease in overall mortality (SMR = 0.54, 95% CI = 0.39–0.73) and non-malignant deaths (SMR = 0.52, 95% CI = 0.35–0.73) compared with the Korean male population.

An analysis of mortality using the ICD-10 major disease classifications revealed that the SMRs for all diseases were significantly lower across ERs and firefighters compared with the Korean male population. Mortality due to circulatory diseases (SMR = 0.38, 95% CI = 0.10–0.98), however, was significantly lower in non-firefighters only.

An analysis of mortality using the ICD-10 middle disease classifications revealed that mortality due to exposure to smoke, fire, and flames (X00-X09) was significantly higher among all ERs (SMR = 3.11, 95% CI = 1.87–4.85), firefighters (SMR = 2.48, 95% CI = 1.33–4.17), and non-firefighters (SMR = 10.50, 95% CI = 3.38–24.51) (Table 2) compared with the Korean male population.

**Table 2:** Mortality (SMR) due to non-malignant disease in emergency responders (ERs) (Reference: Korean men).

Cause of death		All ERs	Firefighters				Non-firefighters
			Total	< 10 years	10–20 years	≥20 years	
	Person-years	377,703	342,233	128,790	148,004	65,079	35,470
All causes	N	485	444	150	137	157	41
	SMR	**0.43**	**0.42**	**0.55**	**0.36**	**0.40**	**0.54**
	95%CI	**0.39–0.47**	**0.38–0.46**	**0.46–0.64**	**0.30–0.42**	**0.34–0.47**	**0.39–0.73**
All non-malignant death	N	308	277	107	89	81	31
	SMR	**0.36**	**0.35**	**0.49**	**0.29**	**0.29**	**0.52**
	95%CI	**0.32–0.40**	**0.31–0.39**	**0.40–0.60**	**0.24–0.36**	**0.23–0.36**	**0.35–0.73**
Infection (A00-B99)	N	10	10	4	3	3	0
	SMR	**0.28**	**0.30**	0.47	**0.24**	**0.24**	-
	95%CI	**0.13–0.51**	**0.14–0.55**	0.13–1.20	**0.05–0.71**	**0.05–0.70**	-
Endocrine diseases (E00-E90)	N	17	16	4	4	8	1
	SMR	**0.44**	**0.44**	0.50	**0.34**	**0.48**	0.45
	95%CI	**0.26–0.71**	**0.25–0.72**	0.14–1.29	**0.09–0.86**	**0.21–0.95**	0.01–2.50
Diabetes (E10-E14)	N	16	15	4	3	8	1
	SMR	0.46	0.45	0.56	**0.28**	0.52	0.50
	95%CI	0.26–0.74	0.25–0.75	0.15–1.44	**0.06–0.82**	0.22–1.02	0.01–2.79
Circulatory diseases (I00-I99)	N	48	44	14	13	17	4
	SMR	**0.27**	**0.27**	**0.37**	**0.24**	**0.25**	**0.38**
	95%CI	**0.21–0.37**	**0.20–0.37**	**0.20–0.62**	**0.13–0.40**	**0.14–0.40**	**0.10–0.98**
Ischemic heart diseases (I20-I25)	N	21	18	7	4	7	3
	SMR	**0.46**	**0.42**	**0.67**	**0.27**	**0.40**	1.03
	95%CI	**0.28–0.70**	**0.25–0.66**	**0.27–1.39**	**0.07–0.68**	**0.16–0.82**	0.21–3.02
Cerebrovascular diseases (I60-I69)	N	18	18	5	5	8	0
	SMR	**0.23**	**0.24**	**0.30**	**0.20**	**0.24**	-
	95%CI	**0.13–0.36**	**0.14–0.38**	**0.10–0.71**	**0.07–0.48**	**0.10–0.46**	-
Respiratory diseases (J00-J99)	N	4	3	0	0	3	1
	SMR	**0.16**	**0.13**	-	-	**0.27**	0.72
	95%CI	**0.05–0.42**	**0.03–0.37**	-	-	**0.05–0.80**	0.01–3.39
Digestive diseases (K00-K93)	N	30	30	7	14	9	0
	SMR	**0.22**	**0.24**	**0.24**	**0.31**	**0.17**	-
	95%CI	**0.15–0.32**	**0.16–0.34**	**0.10–0.49**	**0.17–0.52**	**0.08–0.33**	-
Liver diseases (K70-K77)	N	29	29	6	14	9	0
	SMR	**0.24**	**0.26**	**0.23**	**0.35**	**0.19**	-
	95%CI	**0.16–0.35**	**0.17–0.37**	**0.08–0.50**	**0.19–0.58**	**0.09–0.37**	-
Injury, poisoning, & external causes (S00-T98)	N	171	148	69	48	31	23
	SMR	**0.49**	**0.46**	**0.67**	**0.35**	**0.37**	0.81
	95%CI	**0.42–0.56**	**0.39–0.54**	**0.52–0.85**	**0.26–0.47**	**0.25–0.52**	0.51–1.22
Transport accidents (V01-V09)	N	48	44	25	14	5	4
	SMR	**0.38**	**0.37**	**0.67**	**0.28**	**0.17**	0.39
	95%CI	**0.28–0.50**	**0.27–0.50**	**0.43–0.98**	**0.15–0.47**	**0.05–0.39**	0.11–1.00
Exposure to smoke, fire, & flames (X00-X09)	N	19	14	5	7	2	5
	SMR	**3.11**	**2.48**	2.85	**2.85**	**1.40**	**10.50**
	95%CI	**1.87–4.85**	**1.33–4.17**	0.92–6.66	**1.14–5.87**	**1.16–5.04**	**3.38–24.51**
Intentional self-harm (X60-X84)	N	51	44	21	12	11	7
	SMR	**0.47**	**0.45**	**0.64**	**0.29**	**0.46**	0.76
	95%CI	**0.35–0.62**	**0.33–0.60**	**0.39–0.97**	**0.15–0.51**	**0.23–0.82**	0.30–1.56

*N*, number of case; *SMR*, standardized mortality ratio; *CI*, confidence interval

### SMRs for malignant deaths

Overall cancer mortality was significantly lower across all ERs (SMR = 0.58, 95% CI = 0.50–0.67) and firefighters (SMR = 0.58, 95% CI = 0.50–0.68) compared with the Korean male population. No significant increase in cause-specific cancer mortality was observed across all ERs, firefighters, or non-firefighters compared with the Korean male population (Table 3).

**Table 3:** Mortality (SMR) due to cancer in emergency responders (ERs) (reference: Korean men).

Site of cancer		All ERs	Firefighters				Non-firefighters
			Total	< 10 years	10–20 years	≥20 years	
	Person-years	377,703	342,233	128,790	148,004	65,079	35,470
All	N	177	167	43	48	76	10
	SMR	**0.58**	**0.58**	**0.66**	**0.51**	**0.59**	0.55
	95%CI	**0.50–0.67**	**0.50–0.68**	**0.48–0.89**	**0.38–0.68**	**0.47–0.74**	0.26–1.01
Stomach	N	35	34	11	9	14	1
	SMR	**0.61**	**0.63**	0.89	**0.50**	0.60	0.29
	95%CI	**0.43–0.85**	**0.43–0.88**	0.44–1.59	**0.23–0.95**	0.33–1.00	0.00–1.62
Colorectum	N	13	12	2	5	5	1
	SMR	0.66	0.65	0.46	0.81	0.63	0.83
	95%CI	0.35–1.14	0.34–1.14	0.05–1.67	0.26–1.90	0.20–1.48	0.01–4.59
Liver & intrahepatic bile duct	N	50	50	14	13	23	0
	SMR	0.52	0.55	0.69	0.43	0.58	-
	95%CI	0.39–0.69	0.41–0.73	0.38–1.16	0.23–0.73	0.37–0.87	-
Bronchus & lung	N	28	26	6	7	13	2
	SMR	0.59	0.58	0.69	0.53	0.56	0.82
	95%CI	0.39–0.85	0.38–0.84	0.25–1.48	0.21–1.10	0.30–0.96	0.09–2.96
Leukemia	N	6	6	1	3	2	0
	SMR	0.61	0.66	0.33	0.83	0.81	-
	95%CI	0.22–1.32	0.24–1.44	0.00–1.86	0.17–2.42	0.09–2.91	-
Lymphohematopoietic cancer	N	16	15	4	6	5	1
	SMR	0.89	0.91	0.80	0.96	0.96	0.72
	95%CI	0.51–1.45	0.51–1.50	0.21–2.04	0.35–2.08	0.31–2.23	0.01–4.03

*N*, number of case; *SMR*, standardized mortality ratio; *CI*, confidence interval

### SMRs by employment duration

Overall mortality (N = 150, SMR = 0.55, 95% CI = 0.46–0.64) and mortality due to circulatory disease (SMR = 0.37, 95% CI = 0.20–0.62), digestive system diseases (SMR = 0.24, 95% CI = 0.10–0.49) and external causes (SMR = 0.67, 95% CI = 0.52–0.85) was significantly lower among firefighters employed <10 years compared with the Korean male population. No significant increase in mortality was observed across the major ICD-10 classifications. Overall cancer mortality (SMR = 0.66, 95% CI = 0.48–0.89) was significantly lower among firefighters employed <10 years compared with the Korean male population. No significant increase in cause-specific cancer mortality was observed among firefighters employed <10 years.

Overall mortality (N = 136, SMR = 0.36, 95% CI = 0.30–0.42) was significantly lower in firefighters employed ≥10 to <20 years compared with the Korean male population. No significant increase in mortality was observed across the major ICD-10 classifications. Mortality due to exposure to smoke, fire, and flames (X00-X09) (SMR = 2.85, 95% CI = 1.14–5.87) was significantly higher in firefighters employed ≥10 to <20 years compared with the Korean male population. Overall cancer mortality (SMR = 0.51, 95% CI = 0.38–0.68), as well as mortality due to stomach (SMR = 0.50, 95% CI = 0.23–0.95) and liver cancer (SMR = 0.43, 95% CI = 0.23–0.73), was significantly lower in firefighters employed ≥10 to <20 years compared with the Korean male population.

Overall mortality (N = 157, SMR = 0.40, 95% CI = 0.34–0.47) and cause-specific mortality was similar among firefighters employed ≥20 years compared with firefighters employed ≥10 to <20 years (Tables 2 and 3).

### Adjusted Relative Risk (ARR) of Mortality

All-cause (ARR = 0.68, 95% CI = 0.55–0.85) and non-malignant disease (ARR = 0.69, 95% CI = 0.52–0.90) mortality was significantly lower in firefighters employed ≥10 to <20 years compared with never-firefighters and firefighters employed <10 years. No significant differences in mortality were observed across the major and middle ICD-10 classifications.

All-cause (ARR = 1.46, 95% CI = 1.13–1.89) and non-malignant disease (ARR = 1.65, 95% CI = 1.16–2.33) mortality was significantly higher in firefighters employed ≥20 years compared with the reference group. Overall cancer (ARR = 1.54, 95% CI = 1.02–2.31) and leukemia (ARR = 83.65, 95% CI = 2.21–3,166.29) mortality also were significantly higher in firefighters employed ≥20 years compared with the reference group. Injury, poisoning, and external causes of mortality (S00-T99) (ARR = 3.13, 95% CI = 1.18–5.46) and intentional self-harm (X60-X84) (ARR = 2.57, 95% CI = 1.01–6.64) were also significantly higher in firefighters employed ≥20 years compared with the reference group (Table 4).

**Table 4:** Adjusted relative risk (ARR) of mortality of firefighters (reference: never-firefighters and firefighters employed < 10 years).

	Cause of deaths	N	ARR ^a^ (95%CI)	N	ARR ^b^ (95% CI)
	All causes	137	**0.68 (0.55–0.85)**	157	**1.46 (1.13–1.89)**
Non-malignant diseases	All causes excluding cancer	89	**0.69 (0.52–0.90)**	81	**1.65 (1.16–2.33)**
	Circulatory diseases	13	1.57 (0.72–3.41)	17	1.40 (0.72–2.74)
	Ischemic heart disease	4	1.35 (0.44–4.14)	7	0.93 (0.34–2.51)
	Cerebrovascular diseases	5	1.30 (0.31–5.43)	8	1.87 (0.63–5.59)
	Respiratory diseases	0	-	3	5.89 (0.34–101.13)
	Digestive diseases	14	1.72 (0.69–4.27)	9	1.17 (0.40–3.44)
	Liver diseases	14	2.00 (0.77–5.23)	9	1.42 (0.46–4.38)
Malignant diseases	All cancers	48	0.76 (0.51–1.12)	76	**1.54 (1.02–2.31)**
	Stomach	9	0.63 (0.27–1.50)	14	1.03 (0.44–2.44)
	Colorectum	5	1.40 (0.33–5.87)	5	1.29 (0.27–6.08)
	Liver & intrahepatic bile duct	13	0.78 (0.37–1.66)	23	1.82 (0.85–3.90)
	Bronchus & lung	7	0.71 (0.26–1.96)	13	1.21 (0.46–3.18)
	Leukemia	3	6.54 (0.50–85.12)	2	**83.65 (2.21–3166.29)**
	Lymphohematopoietic diseases	6	1.22 (0.36–4.11)	5	3.26 (0.67–15.80)
Injury, poisoning, & external causes	Injury, poisoning, & external causes	48	0.75 (0.52–1.08)	31	**3.13 (1.80–5.46)**
	Transport accidents	14	0.79 (0.40–1.57)	5	3.18 (0.93–10.84)
	Intentional self-harm	12	0.56(0.28–1.12)	11	**2.57 (1.01–6.64)**

*N*, number of cases; *CI*, confidence interval

^a^ ARRs of firefighters employed ≥10 years to < 20 years

^b^ ARRs of firefighters employed ≥20 years

## Discussion

### Strengths and limitations of the study

The cohort in the present study, which included a high proportion (85%) of active workers, exhibited a large ‘healthy ER effect’ on mortality, as was observed in our previous cancer morbidity study [5]. The Korea NEMA requires individuals employed as ERs to be of high physical fitness (‘Healthy ER selection effect’). Furthermore, ERs are required to maintain physical fitness throughout their employment (‘Healthy ER survivor effect’). Therefore, the healthy worker effect of ERs is greater than that contributed by other workers. This effect is supported by medical check-up data for male ERs (20–29 years old) compared with male non-ERs in the same age range in Korea. The health status (e.g., level of blood pressure, fasting blood sugar, and total cholesterol) and lifestyle factors (e.g., proportion of overweight individuals, prevalence of smokers, and frequency of physical exercise) of ERs were significantly better than those of Korean men [4, 5]. Health status and lifestyle are closely related to morbidity and mortality for several diseases, including cancer, cardiovascular disease, and diabetes. Therefore, it is reasonable to expect that some cause-specific mortality would be lower among ERs compared with the general Korean population [4, 5].

Among the present cohort, 88% of ERs have experience in firefighting due to job rotation. Other ERs within the Korea NEMA also experience similar environmental exposures to that of firefighters. For example, both rescue men and emergency medical aids are present at the scene of a fire. However, the intensity of the exposure is different between firefighters and other ERs, which may dilute the job-related mortality risk between firefighters and other ERs in the present cohort. ERs within the Korea NEMA are also exposed to shift work (in Korea, 24-hours on and off duty without other holidays) and job stress [4, 5].

The small number of mortality cases in the present cohort is a major limitation of this study. Only 485 deaths (128.4 per 100,000 person years) were observed over 16 years of follow-up. Furthermore, some cause-specific mortality types failed to reach statistical significance due to the small number of cases. In our previous cancer morbidity study on this same cohort, we observed statistically significant increases in colo-rectum, kidney, and bladder cancers as well as non-Hodgkin’s lymphoma [5]. However, only 177 cancer deaths were observed, which reduced the ability to detect significant differences in cancer mortality. Therefore, a longer follow-up period is needed in this cohort to obtain enough mortality cases for statistical significance.

Despite the limitations of this study, our aim was to examine morbidity and mortality among ERs other than firefighters. Mortality studies for ERs other than firefighters and police officers are rare. This study therefore provides valuable information on mortality for other ERs such as emergency medical aids and rescue men.

In the current study, we also tried to analyze the SMRs of ERs in reference to the Korean male population (external comparison) and the ARRs of experienced firefighters in reference to non-firefighters (internal comparison) to overcome the ‘healthy ER selection and survivor effect’. Based on our internal comparison, we found that some mortality among firefighters may be work-related.

### Concordance with previous studies

Few previous studies exist on non-cancer mortality in firefighters. In addition, no previous studies exist on mortality among non-firefighting ERs who belong to fire brigades, such as rescue men and emergency medical aids. Therefore, the mortality results in the present study provide valuable information about non-firefighting ERs.

Previous morbidity and mortality studies have reported that cancer, respiratory disease, and cardiovascular disease, including coronary heart disease, among firefighters may be related to occupational exposure. However, associations between occupational exposures and these diseases have been inconsistent.

Recently, the IARC (International Agency for Research on Cancer) Working Group conducted a meta-analysis of studies performed on firefighters and cancer. The results of this meta-analysis suggested that firefighters had a higher risk for non-Hodgkin’s lymphoma as well as prostate and testicular cancers [8]. In our study, however, no significant increase in overall or cause-specific cancer mortality was observed. Overall cancer and leukemia mortality were significantly increased only in firefighters employed ≥20 years compared with non-firefighters and firefighters employed <10 years. Among chemical carcinogens measured at fire scenes, benzene [4, 9–13], 1,3-butadiene [12, 14], and formaldehyde[4, 9, 11, 13–15] have been confirmed as carcinogens that cause lymphohematopoietic cancer. A meta-analysis conducted on 32 cancer morbidity and mortality studies [16] found a potential association between firefighting and leukemia. A significant increase in leukemia observed in the present study may therefore be work-related due to exposure to benzene and other chemicals during firefighting. However only 2 cases of leukemia death were observed among firefighters employed ≥20 years. So, more follow-up needs to assure association between exposure during firefighting job and leukemia death.

Firefighting requires extreme physical exertion with high aerobic demands and static loads. In addition, exposure to carbon monoxide may induce tissue hypoxia and worsen cardiovascular demand. These factors may potentially cause coronary artery disease in on-duty firefighters [17]. For the 10 most recent years, among the 52 deaths that occurred in on-duty Korean ERs, only 2 cases (3.8%) were related to sudden cardiac death [4]. During this period, however, approximately 80 cases were compensated by GEPS due to work-related cardiovascular disease. This statistic indicates, therefore, that most ERs who experienced a cardiovascular event were off-duty and survived. In contrast, among 103 on-duty firefighter deaths in 2008 in the United States, 36 cases (35%) were related to sudden cardiac death [18]. These results suggest that mortality and morbidity rates of cardiac disease among firefighters are inconsistent. Choi [19] reviewed 23 firefighter mortality studies to summarize coronary artery disease in this population. He reported that 11 studies showed positive evidence for an association between firefighting and heart disease, but 12 studies showed no evidence for an association. Choi concluded that there is strong evidence for an overall increased risk of death from heart disease among firefighters. There was insufficient evidence, however, for an increased risk of death from aortic aneurysm or any other heart disease subtype, such as acute myocardial infarction, but some sub-cohort analyses showed significantly increased mortality for circulatory diseases. Another study [20], which looked at mortality among Philadelphia firefighters, found significantly increased mortality due to ischemic heart diseases (SMR = 1.09). A meta-analysis of 17 published firefighter mortality studies from 1959 to 2001[2], which used the SMR as a summary measure, revealed that employment as a firefighter was not associated with all-cause, coronary artery disease, cancer, or respiratory disease mortality. Another study [21] on mortality among professional firefighters in Florida also found no significant increase in all-cause (SMR = 0.57, 95% CI = 0.54–0.60), all-cancer (SMR = 0.85, 95% CI = 0.77–0.94), or cardiovascular disease (SMR = 0.73, 95% CI = 0.65–0.83) mortality among male firefighters. This study found that mortality due to cardiovascular disease (SMR = 2.49, 95% CI = 1.32–4.25) was significantly increased, however, in female firefighters. Our study, in contrast, showed a significant decrease in overall circulatory disease and ischemic heart disease mortality in firefighters, non-firefighters, and all ERs compared with the Korean male population. Further follow-up that excludes the healthy ER effect and includes a more detailed job-exposure analysis is needed to explain the causal relationship between circulatory disease, especially ischemic heart diseases, and firefighting.

Despite the use of a self-contained breathing apparatus, firefighters continue to be at risk for respiratory effects of exposure to irritant chemicals and particulates, especially at overhaul. Severe smoke inhalation can lead to burns in the respiratory tract, pulmonary edema, and acute respiratory distress syndrome. Less severe exposures can cause significant irritant effects, including reactive airway dysfunction syndrome and asthma [17]. Several studies on acute and chronic respiratory disease among firefighters have been conducted [2,13,22–31]. Among these studies, only a few have revealed a significant incidence of chronic respiratory diseases such as chronic obstructive pulmonary diseases and asthma. Most respiratory diseases among firefighters were acute, such as pulmonary edema and decreased pulmonary function test. Few mortality studies on respiratory diseases among firefighters have been conducted. A review of 17 published firefighter mortality studies [2] found no association between respiratory disease mortality and employment as a firefighter. The mortality study among firefighters in Florida [21] also found no significant increase in respiratory disease mortality (SMR = 0.50, 95%CI = 0.35–0.70) among male firefighters. In our present cohort, only 4 deaths due to respiratory disease were observed across all ERs. In addition, two cases of acute lower respiratory infections occurred across all ERs, which represented a significant increase (SMR = 9.33, 95%CI = 1.05–33.67) (data not shown) relative to the Korean male population, but could not be associated with exposure-related excess due to the small sample size. A significant decrease in mortality (2 cases, SMR = 0.21, 95%CI = 0.02–0.77) (data not shown) due to chronic lower respiratory disease was observed in firefighters in the present study, which is consistent with the findings from previous studies. Exposure to chemicals and particulates during firefighting operations should have decreased in recent decades due to the increased use of respiratory protection. Changes in patterns of diseases resulting from such exposures may emerge slowly over decades due to the protracted latency of many of these conditions [17]. A long follow-up for gathering more cases to reach significant statistical power and detailed analysis that considers respiratory protection is therefore needed to identify the association between firefighting and respiratory disease mortality.

ERs experience many work-related injuries. Most previous mortality studies, however, have not identified an association between external causes (injuries) and mortality [20, 21, 32, 33]. Few studies have observed significant increases in mortality due to overall injury [34] and falls [35]. The present study also showed a significant decrease in mortality due to external causes among firefighters and all ERs. Among external causes, only mortality due to smoke, fire, and flame exposure was significantly increased in firefighters and non-firefighters. This result indicates that other ERs, such as rescue men, have died due to smoke, fire, and flames. Our study also showed a significant increase in mortality due to all external causes in firefighters compared with non-firefighters (data not shown). This result indicates that firefighters have more dangerous job exposures than other ERs. In an analysis of compensated fatalities due to external causes from 2004 to 2008 among ERs within the Korea NEMA, the fatality rate of firefighters (3.3 per 100,000 emergency responses) were 5.3 and 83 times higher than those of rescue men (0.6) and emergency medical aid men (0.04), respectively [4].

Previous studies [7,20, 21, 32] have shown lower suicide mortality in firefighters. The present study also showed decreased suicide mortality in firefighters and across all ERs compared with the Korean male population. The proportion of suicide mortality to overall mortality was 10.5% in firefighters (51 suicides among 485 deaths), however. In contrast, the proportion of suicide mortality to overall mortality was 4% among Korean men [4], over the same period. This result suggests an increased need for psychological care in ERs, especially firefighters.

Above mentioned about ‘healthy ER effect’, confounding factors related to mortality and morbidity showed better health index (e.g., low level of blood pressure, fasting blood sugar, and total cholesterol) and lifestyle factors (e.g., proportion of overweight individuals, prevalence of smokers, and frequency of physical exercise) in all ERs comparing to Korean male population. It might affect significantly lower overall- and cause-specific mortality, especially circulatory diseases, among ERs compared with the general Korean population in this study.

## Conclusions

This study investigated mortality among Korean firefighters and other ERs, and demonstrated the importance of addressing issues of selection bias, such as healthy firefighter selection and survivor effects, in analyses of the association between exposure and health effects. A strong healthy firefighter effect resulted in significantly decreased all-cause, cancer, ischemic heart disease, and respiratory disease mortality across all ERs, firefighters, and non-firefighters. These results were similar to previous studies that have been conducted among firefighters in other countries. The interpretation of these findings is hampered, however, by the small number of mortality cases and limited exposure history of individual workers as well as broad job categories that do not contain details about task assignments. Increased all-cause, all cancers including leukemia, and injury, poisoning & external cause mortality in firefighters employed for ≥20 years compared with non-firefighters and firefighters employed <10 years suggests that these mortalities might be related to occupational exposure. Further follow-up is necessary to clarify these findings.
