# Correction: Mortality Due to Malignant and Non-Malignant Diseases in Korean Professional Emergency Responders

**DOI:** 10.1371/journal.pone.0139255

**Published:** 2015-09-25

**Authors:** Yeon-Soon Ahn, Kyoung Sook Jeong

1. There is an error in reference 7. The correct reference is Taeger D, Sun T, Keil U, Straif K. A stand-alone window application for computing exact person-years, standardized mortality ratios and confidence intervals in epidemiologic studies. Epidemiology 2000;11:607–608.
2. In the Methods section, there is an error in the equation in the section titled "Statistical Analysis." The formula is incorrect. Please view the complete, correct formulas and added text here:
  $$P(X≧x|\mu =\mu_{L})=\sum_{k=x}^{\infty }(e^{-\mu_{L}}\;\mu_{L}^{k})/k!≦\alpha /2,$$
  $$P(X≦x|\mu =\mu_{U})=\sum_{k=0}^{x}(e^{-\mu_{U}}\;\mu_{U}^{k})/k!≦\alpha /2$$
  Where X is a Poisson random variable with parameter *μ* of the Poisson distribution, *x* is a observed value of X, and *μ*_*L*_ and *μ*_*U*_ are the values of the lower confidence limit and upper confidence limit of *μ* respectively, and the *α* is 0.05 at 95% CI.
3. A reference is omitted from the last sentence of the first paragraph under the subheading "Statistical Analysis" in the Methods section. The sentence should read: In theory, confidence interval is expressed as following equation at 95% CI (Sahai et al., 1993). The reference is: Sahai H, Khurshid A. Confidence intervals for the mean of a Poisson distribution: A review. Biom J. 1993;35:857–867.
